# ﻿*Akhania*, a new genus for *Salsoladaghestanica*, *Caroxyloncanescens* and *C.carpathum* (Salsoloideae, Chenopodiaceae, Amaranthaceae)

**DOI:** 10.3897/phytokeys.211.89408

**Published:** 2022-10-14

**Authors:** Alexander P. Sukhorukov, Alina V. Fedorova, Maria Kushunina, Evgeny V. Mavrodiev

**Affiliations:** 1 Department of Higher Plants, Biological Faculty, Lomonosov Moscow State University, 119234, Moscow, Russia; 2 Laboratory Herbarium (TK), Tomsk State University, Lenin Ave. 36, 634050, Tomsk, Russia; 3 Tsitsin Main Botanical Garden of Russian Academy of Sciences, 127276, Botanicheskaya St. 4, Moscow, Russia; 4 Department of Plant Physiology, Biological Faculty, Lomonosov Moscow State University, 119234, Moscow, Russia; 5 Florida Museum of Natural History, University of Florida, Gainesville, Florida, 32611, USA

**Keywords:** *Akhania*, Amaranthaceae, Chenopodiaceae, molecular phylogeny, new genus

## Abstract

Genus *Salsola* s.l. was recently split into several genera of different phylogenetic placements within Salsoloideae, but both taxonomic and phylogenetic relationships of some parts of the former broadly defined *Salsola* still need to be clarified. A remarkable example is *Salsolacanescens* nom. illegit. ≡ *Salsolaboissieri*, a taxon with tricky taxonomic history that was only recently transferred to the genus *Caroxylon* (tribe Caroxyleae). *Salsoladaghestanica*, a narrow endemic of Central Dagestan (Russian Federation), was not even included in previous molecular studies of Salsoloideae and therefore still lacks an appropriate estimation of its relationships. Molecular phylogeny constructed here using nuclear and plastid DNA sequence data clearly placed *Salsoladaghestanica* and *Caroxyloncarpathum* as sister taxa and the clade *S.daghestanica*, *Caroxyloncanescens* (*Salsolaboissieri*), *C.carpathum* (*Salsolacarpatha*) as a sister of the monophyletic *Caroxylon*. All three species are distinct from *Caroxylon* from a morphological standpoint. In conclusion, a new genus, *Akhania*, was established for these taxa. The detailed distribution of *Akhaniadaghestanica* is presented for the first time.

## ﻿Introduction

If circumscribed broadly, genus *Salsola* L. encompasses a large number of species, mostly distributed in the steppes, deserts and mountains of Eurasia, northern and southern Africa (e.g., Freitag and Rilke 1997). Comprehensive molecular phylogeny of subfamily Salsoloideae (Akhani et al. 2007) clearly revealed that *Salsola* is widely polyphyletic, and thus the broad circumscription of the genus is of purely historic interest. The members of *Salsola* s.l. must be transferred to numerous reinstated or newly established genera (Akhani et al. 2007, 2016; Rudov et al. 2020). The current system of Salsoloideae (Akhani et al. 2007) is in good congruence with both morphological and biochemical data (Akhani et al. 2007), and today it appears as widely accepted (e.g., Wen et al. 2010; Feodorova and Samigullin 2014; Sukhorukov 2014; Hernández-Ledesma et al. 2015; Sukhorukov et al. 2016, 2019; Mucina 2017, among others).

However, both taxonomic and phylogenetic relationships of some parts of the former broadly defined *Salsola* still need to be clarified. A remarkable example is *Salsolacanescens* (Moq.) Boiss. [nom. illegit., non Pers.], a Western and Central Asian taxon that was recently transferred to the genus *Caroxylon* Moq. (tribe Caroxyleae) (Akhani et al. 2007) but previously was included in Salsolasect.Belanthera Iljin under the name *S.boissieri* Botsch. (Bochantsev 1968). The latter binomial was substituted by the names *Caroxyloncanescens* (Moq.) Akhani (Akhani et al. 2007), *C.boissieri* (Botsch.) Freitag nom. superfl. (Breckle et al. 2013) and *Climacopteracanescens* (Moq.) G.L.Chu (Zhu and Sanderson 2017). However, Sukhorukov et al. (2016) have pointed out that *Caroxyloncanescens* is morphologically different from all other members of *Caroxylon* Thunb. as well as of *Climacoptera* Botsch., and thus, its circumscription with either of these two genera is problematic. So, despite the phylogenetic results of Akhani et al. (2007), the taxonomic placement of *S.boissieri* (former *S.canescens*) remains ambiguous. This ambiguity also applies to *Caroxyloncarpathum* (P.H.Davis) Akhani & Roalson (*Salsolacarpatha* P.H.Davis), a narrow endemic of the Greek Islands that are situated in the Aegean Sea. This species is morphologically very close to *C.canescens*, which was already mentioned by Davis (1953), who considered both taxa within broadly defined *Salsola* (Davis 1953).

Whereas *Salsolacanescens* and *S.carpatha* are eventually considered to be a part of *Caroxylon* (Akhani et al. 2007), *Salsoladaghestanica* (Turcz.) Turcz., a remarkable narrow endemic of Central Dagestan (Eastern Caucasus, Russian Federation), was not even included in the previous molecular studies of Salsoloideae Raf. and therefore still lacks an appropriate estimation of its relationships. This mysterious species grows in the foothills and mountains at elevations of up to 1,200 m a.s.l. It is morphologically well-recognizable due to the striking unique combination of bushy habit, long linear leaves that are not gibbous at base, bracts that are longer than flowers, small wing-like perianth appendages, and anther thecae divided almost to the top. Because of the latter character, *S.daghestanica* was also included in Salsolasect.Belanthera (e.g., Iljin 1936; Bochantsev 1980) and currently this species is preliminarily considered within *Salsola* (Sukhorukov and Akopian 2013). However, this species differs from other *Salsola* in having shrubby vs. annual (rarely subshrubby in *Salsolagriffithii* (Bunge) Freitag and Khani only) life history, soft simple hairs vs. papillae, obtuse vs. mucronate leaf tips, and large vs. inconspicuous anther appendages. Based on the evidence from the external morphology, *Salsoladaghestanica* must undoubtedly be placed within the tribe *Caroxyleae*, not *Salsoleae* (Akhani et al. 2007).

*Salsoladaghestanica* is not mentioned in the study of Tzvelev (1993) who considered sect. Belanthera as a part of the restored genus *Caroxylon* even before the widespread use of molecular methods. Elenevsky (1966) proposed that there is a close relationship between *S.daghestanica* and *S.canescens*, but this proposition still needs to be confirmed and clarified.

Due to the pending taxonomic positions of *Caroxyloncanescens*, *C.carpathum* and *Salsoladaghestanica*, the estimation of the correct phylogenetic and taxonomic relationships of these three taxa within the frameworks of molecular phylogenetics, conventional comparative morphology, and biogeography is the main goal of our study.

## ﻿Materials and methods

### ﻿Taxon sampling

Specimens of *Salsoladaghestanica* were studied in eight herbaria (LE, LECB, MW, MHA, MOSP, MSK, MSKU, RV, RWBG, and WIR). The distribution map (Fig. 3) is based on the original summary of all analyzed herbarium specimens (Appendix 1), as well as on additional data from the GBIF database (GBIF Secretariat 2021), and the studies of Grossheim (1945), Murtazaliev (2009, 2016), and Magomedova et al. (2016). This map was prepared using SimpleMappr online tool (Shorthouse 2010).

### ﻿DNA extraction, amplification and sequencing

The total DNA was extracted from the herbarium leaf tissues using the DNeasy Plant Pro Kit (Qiagen, Germany, https://www.qiagen.com) according to the manufacturer’s protocol. Following Akhani et al. (2007), we used two molecular markers for the molecular phylogenetic analysis: nuclear ribosomal internal transcribed spacer (ITS) and plastid intergenic spacer (IGS) *psb*B-*psb*H. For the amplification of the ITS region we utilized primers NNC–18S10 and C26A (Wen and Zimmer 1996). The *psb*B-*psb*H IGS was amplified utilizing primer psbB-psbH-f and psbB-psbH-r (Xu et al. 2000). The PCR cocktail (20 µL) contained 1.5–2 ng of the total DNA, 5 pmol of each primer, 4 µL of Ready-to-Use PCR Master mix 5× MasDDTaqMIX-2025 containing a “hot-start” SmarTaq DNA polymerase (Dialat Ltd., Moscow, Russia, http://en.dialat.ru), and 13 µL of deionized water. PCR reaction was performed using a MJ Research PTC220 DNA Engine Dyad Thermal Cycler (BioRad, Foster City, CA, United States, https://www.bio-rad.com). For the ITS loci, the PCR profile included the initial DNA denaturation at 94 °C for 3 min and 34 reaction cycles of DNA denaturation at 94 °C for 20 s, annealing primers at 50 °C for 30 s, and the extension of the new strands of the DNA at 72 °C for 40 s, with the final 8 min of the extension at 72 °C. For *psbB-psbH*IGS, the PCR profile included the initial DNA denaturation at 94 °C for 3 min and 33 reaction cycles of DNA denaturation at 94 °C for 30 s, annealing primers at 53 °C for 30 s, and the extension of the new strands of the DNA at 72 °C for 90 s with the final 8 min of the extension at 72 ° C. The PCR products were purified by precipitation in 0.125 M ammonium acetate solution in 70% ethanol (Daniels 2003) and visualized on the 1% agarose gel in 0.5× TBE buffer containing ethidium bromide. All PCR products were sequenced on a 3730 DNA Analyzer (Applied Biosystems, Foster City, CA, USA, https://www.thermofisher.com) at the LLC Syntol, Moscow, Russia (https://www.syntol.ru) using the same primers that were used to amplify both loci.

All sequences were deposited in the GenBank database; the accession numbers of the newly obtained sequences are presented in the Table 1.

**Table 1.** Collection data, collectors, the numbers of isolates and the GenBank accession numbers of newly analyzed samples of *Salsoladaghestanica* and *Caroxyloncanescens*.

Species, isolate	Specimens	GenBank accession numbers: ITS	GenBank accession numbers: *psb*B-*psb*H IGS
*Caroxyloncanescens*, isolate CARC1	Israel, Mt. Hermon, September 2017, O. Fragman-Sapir *s.n*. (MW)	OP364980	OP493534
*Salsoladaghestanica*, isolate SD1	Dagestan, Laksky distr., nr Kamasha vill., 8 Aug 1953; Magomedov *s.n*. (MHA0233906)	ON502720	ON512444
*S.daghestanica*, isolate SD2	Dagestan, Laksky distr., 18 Oct 1953; Magomedov *s.n*. (MHA0233907)	ON502721	ON512445
*S.daghestanica*, isolate SD3	Dagestan, Laksky distr., nr Kamasha vill., 18 Aug 1953; Magomedov *s.n*. (MHA0233905)	ON502722	ON512446

### ﻿Molecular alignment and phylogenetic analysis

The core dataset was reconstructed utilizing the Genbank numbers first published by Akhani et al. (2007); the new sequences of ITS and *psbB-psbH*IGS loci of *Salsoladaghestanica* and *Caroxyloncanescens* (Table 1) were added to the analyses. In total, 141 ITS and 118 *psb*B-*psb*H IGS sequences were analyzed in the present study. The concatenated alignment contained 117 nucleotide sequences.

The ITS and *psbB-psbH*IGS sequences were first aligned separately using MAFFT v. 7 with the strategy L-INS-I (Katoh et al. 2002; Katoh and Standley 2013), manually corrected and concatenated with BioEdit v. 7.0 (Hall 1999). The alignments of the ITS and plastid sequence data were analyzed individually, as well as the combined Supermatrix.

The Maximum Likelihood (ML) analyses of all molecular alignments (Felsenstein 1981) were conducted with RAxML v. 8.2.10 using raxmlGUI beta version 2.0 (Stamatakis 2014; Kozlov et al. 2019; Edler et al. 2021) under the assumptions of GTR + GAMMA model (Stamatakis 2014). ML bootstrap values (Sauermann 1989) were based on 1000 fast replicates (Stamatakis 2014). We visualized the resulting trees in FigTree v.1.4.3 (Rambaut and Drummond 2012) and finally prepared all the figures using the InkScape v.0.48.2 (https://inkscape.org/release/inkscape-0.48/).

As in Akhani et al. (2007), the ITSML tree was rooted relative to *Bienertiasinuspersici* Akhani (DQ499349), *Suaedamaritima* (L.) Dumort. (EF453508), *Suaedacucullata* Aellen (EF453509) [all – Suaedoideae], *Kalidiumcaspicum* (L.) Ung.-Sternb. (EF453444), *Microcnemumcoralloides* (Loscos & J.Pardo) Font Quer (EF453448), and *Salicorniapersica* Akhani (EF453460) [all – Salicornioideae]. To root the ML trees that were based on (a) *psbB-psbH*IGS and (b) concatenated alignments we used *Bienertiasinuspersici*, *Microcnemumcoralloides*, and *Suaedamaritima* (Akhani et al. 2007).

## ﻿Results

### ﻿Molecular phylogeny

The total number of characters in the final ITS alignment was 781, consisting of 379 invariant characters (proportion = 0.485) and 484 variable characters. The total number of characters in the final *psb*B-*psb*H IGS (plastid) alignment was 751, consisting of 159 invariant characters (proportion = 0.211) and 281 variable characters. The total number of characters in the final concatenated alignment was 1,532, consisting of 517 invariant characters (proportion = 0.337) and 741 variable characters.

**Figure 1.**
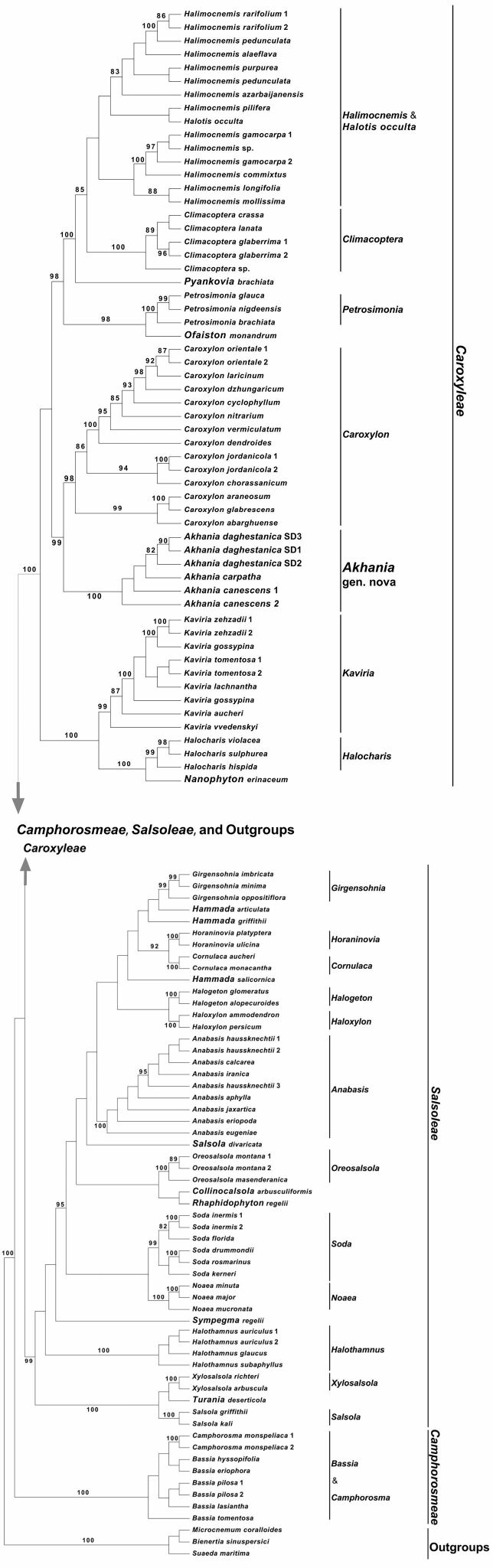
The best tree (–InL= 19360.032213) recovered from the ML analysis (RAxML with GTR + GAMMA) of the ITS plus *psbB-psbH*IGS Supermatrix of Caroxyleae, Salsoleae, Camphorosmeae and outgroups. Numbers above branches indicate ML Bootstrap support values that are equal to or more than 80%. Image: Evgeny V. Mavrodiev.

The ML analysis of ITS dataset resulted in a tree with- InL= 15718.422393 (Suppl. material 1: Fig. S1). The ML analysis of *psb*B-*psb*H IGS dataset resulted in a tree with – InL= 5091.478154 (Suppl. material 2: Fig. S2). The shapes of the obtained trees were softly incongruent, clearly showing the lack of significant character conflict between nuclear and plastid sequence data. The ML analysis of combined dataset resulted a tree with – InL= 19360.032213 (Fig. 1).

The shape of the ML tree that resulted from the analysis of the concatenated matrix is identical to the tree in Akhani et al. (2007), as was expected. Because of this, we limit the description of the results only to the key findings that are related to the major goal of this study: our analysis clearly revealed that *Salsoladaghestanica*, *Caroxyloncanescens* and *Caroxyloncarpathum* are sister taxa forming a clade ‘Akhania’, which appeared to be a highly supported sister (ML BS = 100% and 99%) of the monophyletic genus *Caroxylon* (Fig. 1).

## ﻿Discussion

Salsolasect.Belanthera s.l. included species with the anther’s thecae divided up to their top (Iljin 1936). This character unites the species from the reinstated genus *Caroxylon* Moq. (Tzvelev 1993; Akhani et al. 2007) and a newly established genus *Kaviria* Akhani & E.H.Roalson (Akhani et al. 2007). Bochantsev (1968) selected *Salsolatomentosa* (Moq.) Spach (now *Kaviriatomentosa* (Moq.) Akhani) as a lectotype of this section and excluded from its circumscription all members of the current genus *Caroxylon* (e.g., species with gibbous leaf base). In his later study, Bochantsev (1980) clearly states that sect. Belanthera encompassed the species with non-gibbous leaf base, divided thecae and noticeable anther’s vesicles — the diagnostic characters of the current genus *Kaviria*. However, species that have been described by Bochantsev (1941, 1980) from the relationship of the variable *Salsolaboissieri* (e.g., *S.titovii* Botsch., *S.podlechii* Botsch.) were not accepted in subsequent accounts (Freitag and Rilke 1997; Breckle et al. 2013) and because of this were not included in recent molecular phylogenetic analyses. Similarly, phylogenetic relationships of some taxa that were proposed to be closely related to *Caroxyloncanescens*, namely of S.canescenssubsp.serpenticola Freitag & E.Özhatay (Freitag and Özhatay 1997), and *Salsolaturcica* Yıld., remain unknown. Because of this, the actual taxonomic composition of the clades *Caroxylon* and *Kaviria*, as well as other related groups, still requires further investigation.

An example of these kinds of ambiguous generic placements are two members of a newly described *Akhania* Sukhor. (below), namely *Caroxyloncarpathum* and *C.canescens*. Both of these taxa were previously considered under *Salsola* (as *Salsolacarpatha* and *S.canescens* ≡ *S.boissieri*).

Also, as we have already mentioned above, *Caroxyloncanescens* was recently transferred to *Climacoptera* (as *C.canescens* (Moq.) G.L.Chu). The latter *ad hoc* combination, however, was created without any explanation (Zhu and Sanderson 2017), but all species of *Climacoptera* in its recent circumscription are annuals and also have a decurrent leaf base. This combination of characters is absent in all members of *Akhania*, incl. *A.canescens*. Also, from the molecular phylogenetic standpoint, *Caroxyloncanescens* (*Salsolaboissieri*) is not closely related to *Climacoptera* (Fig. 1).

Despite the fact that, based on the evidence from its external morphology, both *Salsolaboissieri* and *Salsolacarpatha* can be included in the genus *Caroxylon* (Akhani et al. 2007), and *S.daghestanica* resembles the species of *Kaviria*, the unique combination of morphological characters (Table 2) strongly supports the separate placement of all three taxa from either *Kaviria* and *Caroxylon*. A new genus, *Akhania* Sukhor. is established in this study for these three members of the same-name clade. On the molecular tree, the latter is a sister of monophyletic *Caroxylon* (Fig. 1, Suppl. materials 1, 2: Figs S1, S2),

**Table 2.** Morphological differences between *Akhania* and *Caroxylon*.

Character / Taxon	*Akhania*	*Caroxylon* spp.
Hairs	not denticulate	denticulate
Leaf base	neither gibbous nor broadened	± gibbous and broadened
Leaf shape and size	linear, lanceolate or broadly lanceolate, flattened, up to 35 mm long	subulate, not flattened, up to 20 mm or scale-like
Bracts	large, not gibbous, orbicular at base, abruptly (in *A.daghestanica*) diminishing above the base	small, gibbous, orbicular but not abruptly diminishing above the base
Anther appendages	conspicuous	unnoticeable or small
Wing-like appendages on the perianth segments	below the middle of the segments	in the middle part of the segments or absent

## ﻿Taxonomic conclusions

### ﻿ Akhania

Taxon classificationPlantaeCaryophyllalesChenopodiaceae

Sukhor., gen. nov. (Caroxyleae, Salsoloideae).

5CF27BFE-8266-5A96-9794-0B62FE51A4D6

urn:lsid:ipni.org:names:77306620-1

#### Type species.

**Figure 2.**
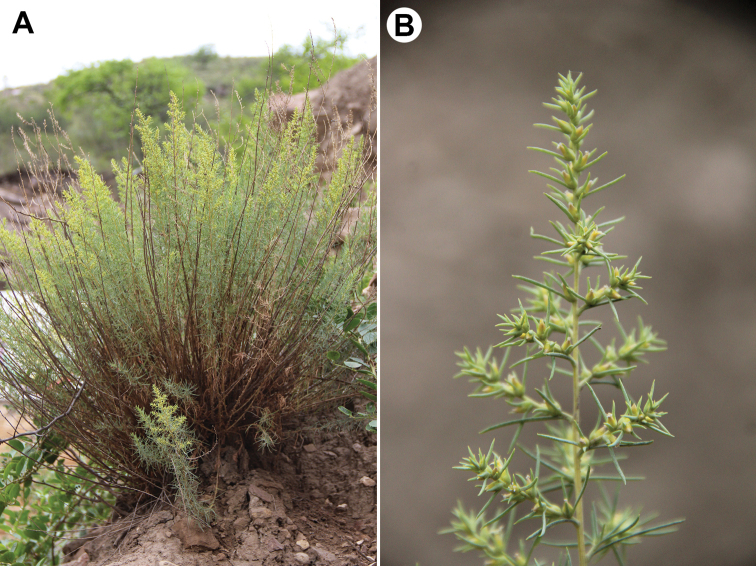
*Akhaniadaghestanica***A** adult plant **B** the upper part of the inflorescence. Photographs by D.S. Shilnikov (Russia, Dagestan, Shamilsky distr., Hebda vill., 14 Jul 2020).

*Akhaniadaghestanica* (Bunge) Sukhor. (Fig. 2).

#### Description.

Subshrubs or small shrubs 20–100 cm tall, with several or numerous stems forming ± bushy habit, glabrous or covered with papillae and tiny caducous simple and smooth (not denticulate) hairs; leaves linear to broadly lanceolate, 5–35 × 1.0–3.0 mm, bright green, glaucous or greyish, covered with appressed simple (partially caducous) hairs, basally not gibbous and not broadened; bracts leaf-like, usually exceeding flowers or equaling, basally orbicular, abruptly (*C.daghestanica*) or continuously (*C.canescens*, *C.carpatha*) diminishing above the base; flowers with two bracteoles smaller than bract; perianth segments 5, glabrous or pubescent, apically obtuse, at fruiting each segment bears wings originated below the middle of each segment; anthers 5, 1.3–3.0 mm long, thecae divided almost to the top, apically with a large (0.8–2.0 mm long) vesicle that is not clearly separated from the thecae; styles shorter than the stigma; seeds with horizontal or vertical embryo position.

#### Etymology.

The new genus is named after Iranian botanist Hossein Akhani.

**Figure 3.**
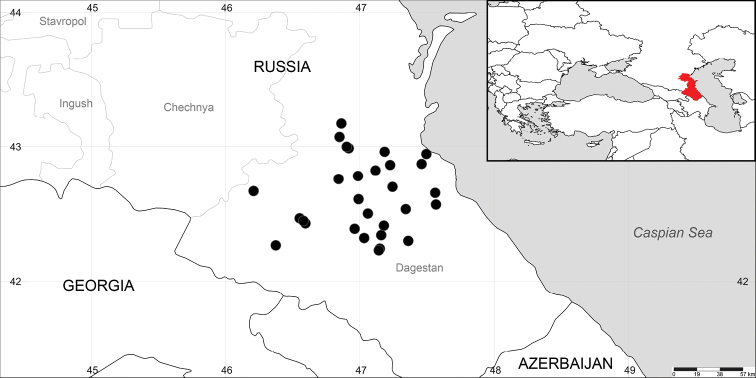
The distribution area of *Akhaniadaghestanica*. The insert map shows the location of Dagestan Republic (colored in red).

The genus consists of three species. The distribution areas of *A.daghestanica* (Dagestan) (Fig. 3), *A.canescens* (Turkey, Iraq, Iran, and Afghanistan) and *A.carpatha* (Aegean Islands) are remarkably disjunctive.

**Figure 4.**
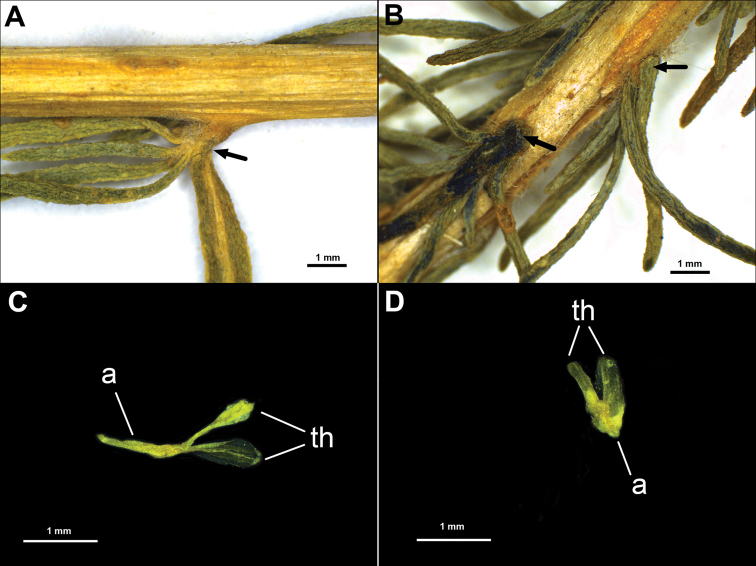
Morphological differences between *Akhania* and *Caroxylon***A** non-gibbous leaf base in *Akhaniadaghestanica***B** gibbous leaf base in *Caroxylonlaricinum***C** anther of *Akhaniadaghestanica* with a large appendage (vesicle) **D** anther of *Caroxylonlaricinum* with a small appendage. Abbreviations: a – anther appendage, th – thecae. Scale bar: 1 mm. Black arrows indicate the leaf base.

*Akhania* differs from the related *Caroxylon* by several remarkable characters or their combinations (Table 2, Fig. 4).

### ﻿1. Akhania daghestanica

Taxon classificationPlantaeCaryophyllalesChenopodiaceae

(Bunge) Sukhor. comb. nov.

0E50E27D-63BA-5C35-AE77-8599F4D12746

urn:lsid:ipni.org:names:77306622-1

≡ Noaeadaghestanica Bunge, Anabas. Rev. [Mèm. Acad. Sci. Pétersb., sér. 7, 4(11)]: 26 (1862).
≡ Salsoladaghestanica (Bunge) Turcz. ex Trautv., Increm. Fl. Phaenog. Ross. 649. 1883; Trautv., Trudy Imp. S.-Peterburgsk. Bot. Sada 9(1): 133 (1884).
≡ Salsoladaghestanica (Bunge) Czern. ex Lipsky, Trudy Imp. S.-Peterburgsk. Bot. Sada 14(2): 295 (1897) isonym.

#### Holotype.

Caucasus orientalis, provincia Daghestan, fl., Patritzky s.n. (KW, isotype – LE!).

#### Distribution.

A local endemic to Dagestan Republic, Russia (Fig. 3).

#### Habitat.

The species is found in open undisturbed habitats, primarily on grassy hills and mountain slopes and screes, at altitudes 500–1200 m a.s.l. It prefers lightly saline and gypsum-enriched soils.

#### Phenology.

Flowering – June–July, fruiting – October.

#### Conservation status.

*Akhaniadaghestanica* has an estimated extent of occurrence of 8,403 km^2^ (which would place the species in the Vulnerable (VU) category according to IUCN (2021)), and an area of occupancy of 112 km^2^ (which would place it in Endangered, EN). Approximately half of the records are dated later than the 1980s (Murtazaliev 2009, 2016; Magomedova et al. 2016; see also Fig. 4, Appendix 1), and the species is likely undercollected, as many Chenopodiaceae are. Therefore, there is no direct evidence of declining population size and fluctuations. However, since the species is found only in natural habitats, it can be threatened by cattle grazing and agriculture. Due to its restricted distribution and possible habitat loss in the future, the species qualifies to be assigned preliminary conservation status of Vulnerable (VU), based on criterion B1 b(iii) of the IUCN Red List categories and criteria (IUCN 2021).

### ﻿2. Akhania canescens

Taxon classificationPlantaeCaryophyllalesChenopodiaceae

(Moq.) Sukhor. comb. nov.

649F482A-CFA8-5DC7-9911-B85C46D6CA81

urn:lsid:ipni.org:names:77306623-1

≡ Noaeacanescens Moq. in DC., Prodr. 13(2): 208 (1849).
≡ Caroxyloncanescens (Moq.) Akhani & Roalson, Int. J. Pl. Sci. 168(6): 947 (2007).
≡ Salsolacanescens (Moq.) Boiss. Fl. Orient. [Boissier] 4: 963 (1879), nom. illegit., non Pers. (1805).
≡ Salsolaboissieri Botsch., Bot. Zhurn. 53: 1442 (1968).
≡ Caroxylonboissieri (Botsch.) Freitag, Vasc. Pl. Afghanistan: 264 (2013), nom. superfl.
≡ Climacopteracanescens (Moq.) G.L.Chu in Chu & Sanderson, Gen. New Evol. System World Chenopod.: 312 (2017).

#### Lectotype

**(Bochantsev 1968)**: [Iraq] In cacumine m. Gara Kurdist. orientem versus frequens, 1843, *Th. Kotschy 346* (LE! isolectotypes G! H, JE, K000899548! P, W0046462!).

#### Distribution.

This species is distributed across Irano-Turanian Region (Freitag and Rilke 1997 as *Salsolacanescens*).

#### Habitat.

The slopes of hills, frequently screes.

#### Phenology.

Flowering – July–August, fruiting – October–November.

#### Conservation status.

The taxonomic composition of this species is still insufficiently studied. Therefore, its current conservation status cannot be properly evaluated.

### ﻿3. Akhania carpatha

Taxon classificationPlantaeCaryophyllalesChenopodiaceae

(P.H.Davis) Sukhor. comb. nov.

22940096-7CE6-56A2-91FA-F44DF8E46BC8

urn:lsid:ipni.org:names:77306624-1

≡ Salsolacarpatha P.H.Davis, Notes Roy. Bot. Gard. Edinburgh 21: 139 (1953).
≡ Caroxyloncarpathum (P.H.Davis) Akhani & Roalson, Int. J. Pl. Sci. 168(6): 947 (2007).

#### Holotype.

Greece, Karpathos [Island], Vurgunda (NW of Olymbos), at 5–20 m alt., on calcareous sea rocks with *Galiumcanum*, 24 Jul 1950, P.H. Davis 18025 (sheet I – K000899552! Sheet II – K000899553! isotype – E00279875!).

#### Distribution.

This species is localized in three Greek islands situated in the Aegean Sea: Crete, Karpathos, Kyklides and adjacent islets (Davis 1953; Christodoulakis et al. 1990; Strid and Tan 1997).

#### Habitat.

Rocks, usually calcareous.

#### Phenology.

Flowering – July–August, fruiting – October–November.

#### Conservation status.

Not evaluated yet, but likely Vulnerable VU (IUCN 2021).

### ﻿Key to the species

**Table:** 

1	Shrub up to 1 m high; bracts abruptly diminishing from the orbicular base, leaves linear, perianth glabrous, wings at fruiting white or yellowish. Endemic of E Caucasus (Dagestan, Russian Federation)	***A.daghestanica***
–	Subshrubs up to 50 cm; bracts continuously diminishing from the orbicular base, leaves ± flattened, perianth pubescent, wings reddish, but turning into brown at dissemination. Species distributed outside of E Caucasus	**2**
2	Leaves linear to lanceolate. Irano-Turanian Region (Turkey, Iraq, Iran, and Afghanistan)	***A.canescens***
–	Leaves broadly lanceolate or narrowly oblong. Islands of the Aegean Sea	***A.carpatha***

## Supplementary Material

XML Treatment for Akhania

XML Treatment for Akhania daghestanica

XML Treatment for Akhania canescens

XML Treatment for Akhania carpatha

## ﻿Appendix 1

**Specimens used for the mapping of *Akhaniadaghestanica* (other records were obtained from Grossheim 1945; Murtazaliev 2009; Magomedova et al. 2016; GBIF Secretariat 2021).**

**Russia. Dagestan Rep.**: [Kizilyurtovsky distr.] Chiryurt vill., 4 Jul 1891, *V. Lipsky s.n*. (LE); [Laksky / Levashinsky distr.] between Kumukh & Tsudakhar vill., 3800 ft, 23 Aug 1898, *Th. Alexeenko s.n*. (LE); [Untsukulsky distr.] nr Gumry vill., 25 May 1901, *Th. Alexeenko 13056* (LE); Temirkhan-Shura [Buynaksk], 3 Oct 1913, *N. Pastukhov exs. 170* (LE, MW0663693); [Gunibsky distr.] nr Choh vill., 16 Oct 1914, *D. Bubaev s.n*. (LE); [Untsukulsky distr.] Arakani gorge, 28 Aug 1927, *A. Poretsky s.n*. (LE); Kakhibsky distr., 26 Jul 1931, *G. Stupnikov 660* (MW0663694); [Buynaksky distr.] nr Nizhny Dzhengutay vill., 2 Aug 1935, *E. Schiffers s.n*. (LE); [Buynaksky distr.] nr Kapchugay vill., 1931, *E. Schiffers 42* (LE); [Kizilyurtovsky distr.] nr Chiryurt vill., 9 Sep 1936, *M. Iljin & E. Iljina 90* (LE); nr Makhachkala town, Tarki vill., 22 Jul 1953, *Ya. Prokhanov 1146* (LE); Laksky distr., nr Kamasha vill., 18 Aug 1953, *Magomedov s.n.* (MHA0233905); Untsukulsky distr., 29 Aug 1953, *Ya. Prokhanov & N. Cheldyshev 372* (LE); [nr Makhachkala town] Kukurtau gorge, 10 Aug 1956, *Ya. Prokhanov 137* (LE); Buynaksky distr., nr Dubki vill., 8 Aug 1981, *Yu. Menitsky & al. 437* (LE); [Buynaksky distr.] nr Chirkey Water Reservoir, 10 km SE of Dubki vill., 11 Jul 2013, *A.S. Zernov 8135* (MW0663692); Shamil’sky distr., Khebda vill., 42.451562 N 46.560036 E, 14 Jul 2020, *D. Shilnikov* (pers. obs., see also Fig. 2).

## Supplementary materials

Supplementary material 1 Figure S1 Data type Image Explanation note The best ML tree (–InL= 15718.422393) recovered from the ML analysis (RAxML with GTR + GAMMA) of the ITS matrix of Caroxyleae, Salsoleae, Camphorosmeae and outgroups. Image: Alina V. Fedorova. https://binary.pensoft.net/file/756830This dataset is made available under the Open Database License (http://opendatacommons.org/licenses/odbl/1.0/). The Open Database License (ODbL) is a license agreement intended to allow users to freely share, modify, and use this Dataset while maintaining this same freedom for others, provided that the original source and author(s) are credited.Alexander P. Sukhorukov, Alina V. Fedorova, Maria Kushunina, Evgeny V. Mavrodiev

Supplementary material 2 Figure S2 Data type Image Explanation note The best ML tree (–InL= 5091.478154) recovered from the ML analysis (RAxML with GTR + GAMMA) of the *psb*B-*psb*H IGS matrix of Caroxyleae, Salsoleae, Camphorosmeae and outgroups. Image: Alina V. Fedorova. https://binary.pensoft.net/file/756831This dataset is made available under the Open Database License (http://opendatacommons.org/licenses/odbl/1.0/). The Open Database License (ODbL) is a license agreement intended to allow users to freely share, modify, and use this Dataset while maintaining this same freedom for others, provided that the original source and author(s) are credited.Alexander P. Sukhorukov, Alina V. Fedorova, Maria Kushunina, Evgeny V. Mavrodiev
